# Phytoplankton Community Response to Environmental Factors along a Salinity Gradient in a Seagoing River, Tianjin, China

**DOI:** 10.3390/microorganisms11010075

**Published:** 2022-12-27

**Authors:** Xuewei Sun, Huayong Zhang, Zhongyu Wang, Tousheng Huang, Hai Huang

**Affiliations:** Research Center for Engineering Ecology and Nonlinear Science, North China Electric Power University, Beijing 102206, China

**Keywords:** river-estuary ecosystem, abiotic factors, seasonal variations, phytoplankton composition, community diversity, salinity

## Abstract

A river-estuary ecosystem usually features a distinct salinity gradient and a complex water environment, so it is enormously valuable to study the response mechanism of living organisms to multiple abiotic factors under salinity stress. Phytoplankton, as an important part of aquatic microorganisms, has always been of concern for its crucial place in the aquatic ecosystem. In this study, phytoplankton data and 18 abiotic factors collected from 15 stations in Duliujian River, a seagoing river, were investigated in different seasons. The results showed that the river studied was of a Cyanophyta-dominant type. Salinity (SAL) was the key control factor for phytoplankton species richness, while water temperature (WT) was critical not only for species richness, but also community diversity, and the abundance and biomass of dominant species. Apart from WT, the abundance and biomass of dominant species were also driven by total nitrogen (TN), nitrate (NO_3_^−^), pH, and water transparency (SD). Moreover, total dissolved phosphorus (TDP), pH, and chemical oxygen demand (COD) were crucial for community diversity and evenness. The bloom of dominant species positively associated with TDP led to lower diversity and evenness in autumn. In addition, when available nitrogen was limited, *Pseudoanabaena* sp. could obtain a competitive advantage through the N_2_ fixation function. Increased available nitrogen concentration could favor the abundance of *Chlorella vulgaris* to resist the negative effect of WT. The results show that *Oscillatoria limosa* could serve as an indicator of organic contamination, and nutrient-concentration control must be effective to inhibit *Microcystis* bloom. This could help managers to formulate conservation measures.

## 1. Introduction

River-estuary ecosystems are considered to be the most productive and dynamic aquatic ecosystems on earth [1,2,3], and have unique physical and chemical properties resulting from the collective influences of upstream freshwater outflow and downstream seawater intrusion. Natural stresses caused by temperature, salinity, sunlight, and dissolved oxygen have been reported to play a crucial role in the biodiversity of river-estuary ecosystems [4,5,6]. Additionally, about 60% of the world’s human population is concentrated near the coasts [7], and the percentage continues to rise. Therefore, river-estuary ecosystems are also inevitably affected by increasing and intensive anthropogenic activities (e.g., urbanization, industrial development, agricultural and aquacultural activities) and their subsequent pressures (e.g., chemical pollution, eutrophication, and harmful algal blooms) [6,8,9]. A deep understanding of which and how environmental factors drive biodiversity variations is imperative not only for environmental managers to obtain scientific information, but also for researchers to gain insight into the river-estuary ecosystem.

Phytoplankton, an essential part of aquatic microorganisms and the major primary producer in aquatic environments, has a crucial place in the food chain and plays an irreplaceable role in energy flow and material cycling [10,11,12]. Numerous studies on phytoplankton communities have demonstrated that phytoplankton-community characteristics such as species richness, community diversity, and community evenness can be driven by many abiotic factors (e.g., water temperature, pH, sunlight, nutrient concentration) and biological factors (e.g., competitive exclusion effect, grazing effect) [11,13,14,15,16,17]. Among the studies on the effects of abiotic factors on phytoplankton, temperature is one of the environmental factors that has received the most attention. At the global scale, temperature could influence phytoplankton growth rates and lead to phytoplankton diversity variations [18], while in microcosm experiments, increased temperature resulted in species loss, biomass decline, and abundance reduction [19,20,21]. In contrast, other studies have found positive or weak effects of temperature on phytoplankton [22,23,24]. Besides this, nutrients have always been the focus of phytoplankton research, and they should be ascribed much responsibility for algal blooms [3,7,25]. Studies have proved that phytoplankton community, species richness, community diversity, and species abundance (e.g., cyanobacteria bloom) are all closely related to nutrient concentrations [4,23,26,27]. In recent years, the impact of salinity on phytoplankton has received much attention. Previous studies revealed that enhanced osmotic pressure induced by increasing salinity is a major cause of phytoplankton changes. For example, some freshwater species fail to overcome the physical constraints of increased osmotic pressure, or some species are outcompeted by species less affected by osmotic pressure [20,21,28]. It is generally considered that salinity is a limiting factor of phytoplankton in coastal areas [29,30,31] and high salinity lakes [11,20,32]. However, few studies have focused on the interactions between environmental factors and phytoplankton communities in river-estuary ecosystems [3,33], which have complex environmental conditions and pronounced salinity gradients.

Duliujian River is the largest river in the southern region of the Haihe River plain. It is an artificial river that links canals, wetlands, reed lakes, and reservoirs on the natural platform and is subject to complex environmental factors caused by intensive anthropogenic activities [34,35,36]. The normally closed estuarine sluice has greatly reduced the impact of seawater intrusion, while the whole river still exhibits a distinct seasonal and spatial salinity gradient. Thus, it was selected as the research object to study the mechanisms between environmental factors and the phytoplankton community under salinity stress.

In this study, phytoplankton data and 18 abiotic factors collected from 15 stations in the river-estuary ecosystem of the Duliujian River were monitored and analyzed in different seasons. The aims of this study were to (i) evaluate the seasonal variations in phytoplankton species composition; (ii) identify the main driving factors affecting the seasonal variations of phytoplankton community characteristics (species richness, community diversity, and community evenness) along the salinity gradient; (iii) identify the response of dominant species to environmental factors along the salinity gradient.

## 2. Materials and Methods

### 2.1. Study Area

**Figure 1:**
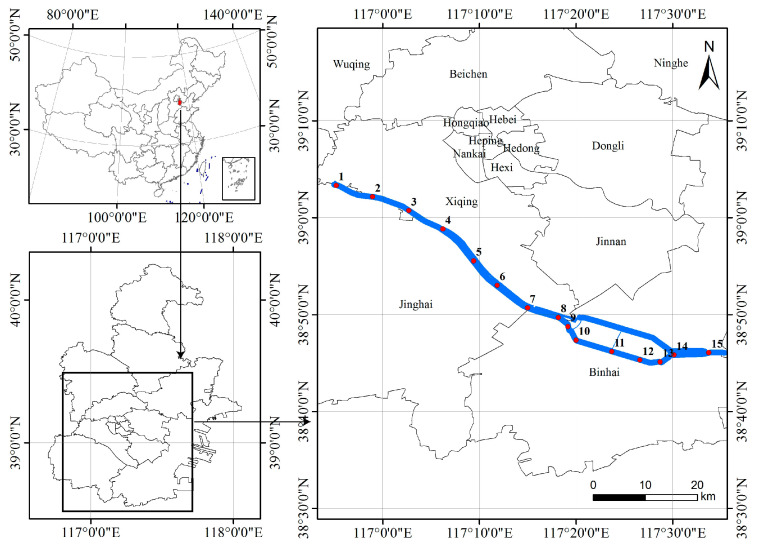
Location map of studied area and sampling stations. Red dots are sampling station.

The Duliujian River, located in Tianjin City (39°3′20′′–38°46′4′′ N, 116°55′10′′–117°33′44′′ E), China, is the largest river in the lower reaches of the southern Haihe River Basin. It flows from the conjunction of the Daqing River and the Ziya River to the Bohai Bay (Figure 1) and covers a catchment area of 3737 km^2^ with a total length of 70.14 km and a maximum width of 1 km in the upper and middle reaches. The river basin belongs to the warm temperate zone with a semi-humid continental monsoon climate, so it has a distinct seasonal environment, windy and dry in spring, hot and rainy in summer, moderate temperature in autumn, and cold in winter. The annual average temperature in this area ranges from 12 °C to 15 °C, and precipitation is concentrated from June to September, with an annual average rainfall of about 571 mm [36]. The river’s seagoing nature makes it susceptible to saline intrusion. As a result, the entire river exhibits a distinct salinity gradient that, along with other abiotic environments, affects the survival, growth, and reproduction of aquatic organisms.

### 2.2. Sample Analysis

As it is shown in Figure 1, 15 sampling stations were set along the whole river considering natural conditions and human activities. Water samples and phytoplankton samples were collected from the surface water at the sampling station quarterly during the period from September 2017 to June 2018, representing the water environment and phytoplankton community in summer, autumn, winter, and spring.

Three parallel water samples were collected at each sampling station, with a 1.5 volume of water per sample, using a polymethylmethacrylate sampler, and stored in polyethylene plastic bottles. Following this, the water samples were placed in insulated boxes and transported to the laboratory within eight hours for further analysis. The samples’ preservation and handling were all performed per relevant national industry standards and guidelines. Some water environmental factors were measured in the suite, such as water temperature (WT), water depth (WD), dissolved oxygen (DO), water transparency (SD), salinity (SAL), pH, and oxidation-reduction potential (ORP), while others were tested in the laboratory, such as total phosphorus (TP), total nitrogen (TN), total dissolved phosphorus (TDP), total dissolved nitrogen (TDN), ammonium nitrogen (NH_4_^+^), nitrate (NO_3_^−^), nitrite (NO_2_^−^), orthophosphate (PO_4_^3−^), turbidity (TUR), and chemical oxygen demand (COD). In the laboratory, the water samples were directly analyzed for TN, TP, COD, and TUR, while the remaining samples filtered through 0.45 μm filter membranes were analyzed for the other nutrient indexes. All water environmental factors were measured in accordance with relevant standards (Supplementary Table S1).

Three parallel phytoplankton samples, 1 L of water per sample, were collected into polyethylene bottles at the 0.5 m surface water depth, and each sample was immediately fixed with 10 mL Lugol’s solution. In the laboratory, phytoplankton samples were stored in glass containers for 24 h, and then the upper section of the solution was siphoned using a rubber hose. For each sample, the remaining 50 mL of sample sediment was transferred into a polyethylene bottle, and preserved by adding formaldehyde (4% *v*/*v*). After 24 h, it was condensed to 30 mL by sucking away the supernatant liquid with a pipette. Following this, the pretreated samples were well packaged and transferred to the Center of Monitoring and Scientific Research of Ecology and Environment, Administration of Ecology and Environment of Haihe Basin and Beihai Area, MEE, to identify phytoplankton species and quantify phytoplankton abundance and biomass. It is worth noting that taxonomical species/taxa of phytoplankton were given under the old nomenclature, but to avoid confusion, they have been updated with new names according to the Algaebase database, added in parentheses after the old name.

### 2.3. Data Analysis

The number of species was considered as the species richness. The dominant species of phytoplankton were determined based on the dominance value (*Y*) of each species [37], as shown in Equation (1). Furthermore, the phytoplankton diversity (*H*) was calculated according to the Shannon–Wiener index [37,38], as shown in Equation (2). The phytoplankton evenness (*J*) was calculated according to Pielou’s evenness index [39,40], as shown in Equation (3).

(1) $$Y=\left(\frac{n_{i}}{N}\right)*f_{i}$$

(2) $$H=-\overset{S}{\underset{i=1}{\sum }}\frac{n_{i}}{N}\ln\left(\frac{n_{i}}{N}\right)$$

(3) $$J=\frac{H}{\ln S}$$

where $n_{i}\;$ is the number of individuals of species i within a given area, N is the number of total individuals of all species, $f_{i}$ is the occurrence frequency of species i, and S is the total species number. Species were considered dominant during the sampling period when the dominance value Y was higher than 0.02 [12,37].

Before statistical analysis, all species data and water environmental factors (except for pH) were log_10_(x + 1) transformed to eliminate dimensional differences. The Kruskal–Wallis nonparametric test was performed on the data to assess significant seasonal discrepancies in environmental factors and phytoplankton community indexes (base package, R, v.4.1.2, R Core Team, Vienna, Austria), considering *p* values < 0.05. Species richness, Shannon–Wiener index, and Pielou’s evenness index were calculated and graphed using ‘ggplot2’ and ‘ggpubr’ packages in R (v.4.1.2).

Non-metric multidimensional scaling (nMDS) was performed based on Bray–Curtis dissimilarity calculated for the species composition of the phytoplankton community (vegan package, R, v.4.1.2). Different colored and shaped points represented different seasons, and the ellipses of the confidence interval were set at the 80% level. Following this, an analysis of similarities (ANOSIM) was run to detect the significant differences (*p* < 0.05) among the phytoplankton communities in different seasons (vegan package, R, v.4.1.2).

Redundancy analysis (RDA, vegan package, R, v.4.1.2) was used to analyze the responses of the phytoplankton community (including abundance and biomass of top 10 dominant phytoplankton species selected according to the dominance values Y, species richness, community diversity H, and community evenness J) to environmental factors. Prior to RDA, detrended correspondence analysis (DCA) was performed to determine whether linear or unimodal ordination methods should be applied. In this study, all DCA results were <4; thus, RDA was the most robust model to analyze the relationships between the phytoplankton community and environmental factors. The forward selection procedure with Monte Carlo permutation tests (999 permutations) was carried out to identify the driving environmental factors of the phytoplankton community variance (function ordiR2step, adespatial package, R, v.4.1.2). Following this, the filtered environmental factors were tested by the variance inflation factor (VIF). The environmental factors with higher VIF values (VIF > 10) or higher correlation coefficients (r > 0.8) were sequentially removed from the model to eliminate collinearity between the selected factors, and then VIF values were recalculated. This procedure was repeated until all the VIF values were smaller than 10. Subsequently, the Mantel test was performed using the Mantel function to test the significance of the relationship between the phytoplankton community and the filtered environmental factors. Finally, the validity and significance of RDA results were analyzed by the Monte Carlo permutation tests (999 permutations), and the results were significant when *p* < 0.05. In addition, the Monte Carlo permutation tests (999 permutations) with Bonferroni adjustment were employed to test whether the first two constraint axes were meaningful at *p* < 0.05 level.

## 3. Results

### 3.1. Environmental Factors

As shown in Table 1, the Kruskal–Wallis test showed statistically significant seasonal variations (*p* < 0.05) for all environmental factors. As a critical water environmental factor in seagoing rivers, SAL has often been of interest to researchers [11,21,29]. In this study, it ranged from 4.42 to 20.73 ppt in autumn, 3.62 to 29.57 ppt in winter, 1.61 to 5.18 ppt in spring, and 1.61 to 5.18 ppt in summer. The maximum and minimum values of SAL, 33.2 ppt and 0.77 ppt, occurred at site 14 (summer) and site 3 (summer), respectively. In terms of pH, the values at all the sampling sites were greater than 8.00 in different seasons, indicating that the water in the study area was slightly alkaline all year round. The recorded maximum WT, 29.27 °C, was at site 15 in summer, while the minimum one is 1.73 °C, at site 13 in winter. DO, with the lowest concentration (3.35 mg/L) in autumn, exhibited great fluctuation in each season. ORP presented the oxidation reducibility of water, ranging from 70.47 (observed in summer) to 221.93 mv (observed in spring). TUR was stable in winter, spring, and summer, with mean values of 15.41 ± 5.57 NTU, 20.82 ± 6.44 NTU, and 27.37 ± 13.96 NTU, respectively, while in autumn, it variated considerably between 7.85 to 77.28 NTU. SD, as the water transparency indicator, showed a significant seasonal variation, with a maximum value of 98.83 cm in autumn and a minimum value of 27 cm in summer. WD had the same change trend in each season, with a slightly higher mean value in winter than in the other seasons. COD varied between 9.19 to 13.83 mg/L, and the mean concentration of 11.97 ± 1.24 mg/L in spring was slightly higher than that in the other three seasons (Supplementary Table S2).

Moreover, TN had a higher mean concentration of 4.59 ± 0.26 mg/L in spring, though it had a similar trend across seasons. TDN was 1.19 ± 0.44 mg/L in autumn, 2.20 ± 0.4 mg/L in winter, 2.88 ± 0.4 mg/L in spring, and 2.13 ± 1.83 mg/L in summer, while the highest concentration of 7.03 mg/L. TP, ranging from an annual maximum concentration of 0.77 mg/L to a minimum value of 0.07 mg/L, fluctuated significantly in summer, but it was much more stable in spring and winter. TP in spring was slightly higher than in winter, while TDP exhibited a different seasonal change, being obviously lower in spring than in winter. The range of TDP was 0.06–0.39 mg/L in autumn, 0.07–0.23 mg/L in winter, 0.05–0.11mg/L in spring, and 0.00–0.50 mg/L in summer, while the maximum value of 0.39 mg/L was observed in autumn. PO_4_^3−^ had a similar trend with TP in each season, with mean concentrations of 0.23 ± 0.07, 0.11 ± 0.04, 0.02 ± 0.02, and 0.10 ± 0.16, corresponding to the study seasons. DIN showed similar change trends in autumn and summer with mean concentrations of 0.29 ± 0.10 mg/L and 0.30 ± 0.16 mg/L, respectively, while its values in spring and winter, respectively, ranging from 0.70–2.37 mg/L and 0.67–2.34 mg/L, were higher than in the other two seasons.

### 3.2. Phytoplankton Community

During the study period, a total of 131 species of phytoplankton belonging to 63 genera and six phyla were identified, among which 58 species from 30 genera of Chlorophyta accounted for 44.27%. Following Chlorophyta, Cyanophyta, with 27 species derived from 11 genera, accounted for 20.61%, and Bacillariophyta, which had 26 species derived from 12 genera, accounted for 19.85%. In addition, 13 species belonging to four genera in Euglenophyta accounted for 9.92%, while four species from Cryptophyta and three species from Dinophyta accounted for 3.05% and 2.29%, respectively.

**Figure 2:**
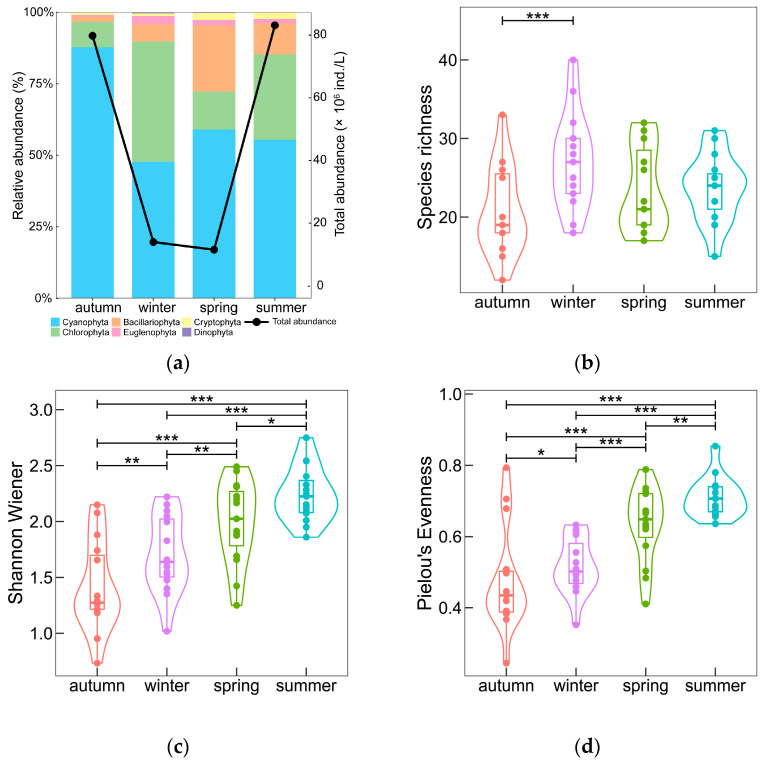
Seasonal variations of phytoplankton abundance and comparisons of the number of species, Shannon–Wiener index, and Pielou’s evenness among different seasons. (**a**) Relative abundance and total abundance of phytoplankton, where the bar stack diagram indicates relative abundance and point and line indicate total abundance; (**b**) Comparison of the number of species; (**c**) Comparison of Shannon–Wiener index; (**d**) Comparison of Pielou’s evenness. “***” denotes *p* < 0.01, while “**” denotes *p* < 0.05 and “*” *p* < 0.1.

The total abundance of phytoplankton fluctuated wildly between seasons, with much larger abundances in autumn and summer than in winter and spring, while Cyanophyta had the highest relative abundances in all four seasons (Figure 2a). Cyanophyta was the most dominant phylum in autumn, taking the largest proportion, 87.78%, with comparatively small changes of relative abundance from 47.68% (winter) to 59.00% (spring) in the other three seasons. With the decline of Cyanophyta from autumn to winter, the relative abundance of Chlorophyta and Bacillariophyta grew significantly, with Chlorophyta reaching its annual peak relative abundance of 42.13%. The proportion of Bacillariophyta gradually increased from its annual minimum in autumn to its annual maximum in spring (23.13%), when it became one of the dominant species, before decreasing to 10.90% in summer. As for Euglenophyta, Cryptophyta, and Dinophyta, the total relative abundance varied between 4.65% (spring) and 1.57% (autumn).

Different combinations of dominant species occurred in different seasons (Table 2). In autumn, *Microcystis* sp. (*Y* = 0.44) from Cyanophyta was the main dominant species, as it showed up in all sample stations with a large proportion of total abundance of 44.31%, whereas the relative proportion of *Chroococcus* sp. (*Y* = 0.07) from Cyanophyta was only 6.69%, though it also appeared in all sample stations. The other dominant species were *Microcystis incerta* Lemmermann (*Aphanocapsa incerta* (Lemmermann) G. Cronberg and Komárek) (*Y* = 0.08) and *Microcystis marginata* (Meneghini) Kützing (*Y* = 0.02) from Cyanophyta, and *Chlorella vulgaris* Beijerinck (*Y* = 0.05) from Chlorophyta. In winter, only two species qualified for *Y* > 0.02, *M. marginata* (*Y* = 0.31) and *C. vulgaris* (*Y* = 0.37), which represented 31.35% and 37.32% of the total abundance, respectively. However, *Cyclotella catenata* (Brun) H. Bachmann from Bacillariophyta, *Pediastrum boryanum* (Turpin) Meneghini (*Pseudopediastrum boryanum* (Turpin) E. Hegewald) and *Tetraëdron trigonum* (Nägeli) Hansgirg from Chlorophyta, all had very high occurrence frequency (>93.33%), whereas they failed to become the dominant species as a result of a lower relative proportion of abundance. *Pseudoanabaena* sp. (*Y* = 0.31), *Oscillatoria limosa* C. Agardh ex Gomont (*Y* = 0.20) from Cyanophyta, and *Cyclotella* sp. (*Y* = 0.11) from Bacillariophyta were the dominant species in spring. However, *Oscillatoria agardhii* Gomont (*Planktothrix agardhii* (Gomont) Anagnostidis and Komárek), *Microcystis flos-aquae* (Wittrock) Kirchner from Cyanophyta, and *Cryptomonas ovata* Ehrenberg from Cryptophyta were observed at more than 93.33% of the sample stations with a relative abundance below 2%. For summer, the dominant species were *Pseudoanabaena* sp., *O. limosa*, *M. flos-aquae*, and *O. agardhii* from Cyanophyta, with dominance values of *Y* = 0.25, *Y* = 0.17, *Y* = 0.04, and *Y* = 0.02, respectively. Besides this, there were *C. vulgaris* (*Y* = 0.13) and *Westella* sp. (*Y* = 0.05) from Chlorophyta, as well as *Cyclotella* sp. (*Y* = 0.06) from Bacillariophyta, with 100% occurrence frequency.

Species richness, Shannon–Wiener index (H), and Pielou’s evenness (J) all had distinctly seasonal variations evidenced by the Kruskal–Wallis test, with *p* < 0.05, *p* < 0.001 and *p* < 0.001, respectively (Table 1). The species richness showed significant difference between autumn and winter (*p* < 0.01), while phytoplankton community diversity increased gradually from autumn to summer according to the pairwise comparisons result of the Shannon–Wiener index, and so did community evenness according to Pielou’s evenness index (Figure 2b–d). The maximum species richness, 73, occurred in spring, and the minimum of 63 was in autumn. The community diversity ranged from 1.41 to 2.24 with an annual average of 1.84, while the community evenness varied from 0.47 to 0.71 with an annual average of 0.58.

**Figure 3:**
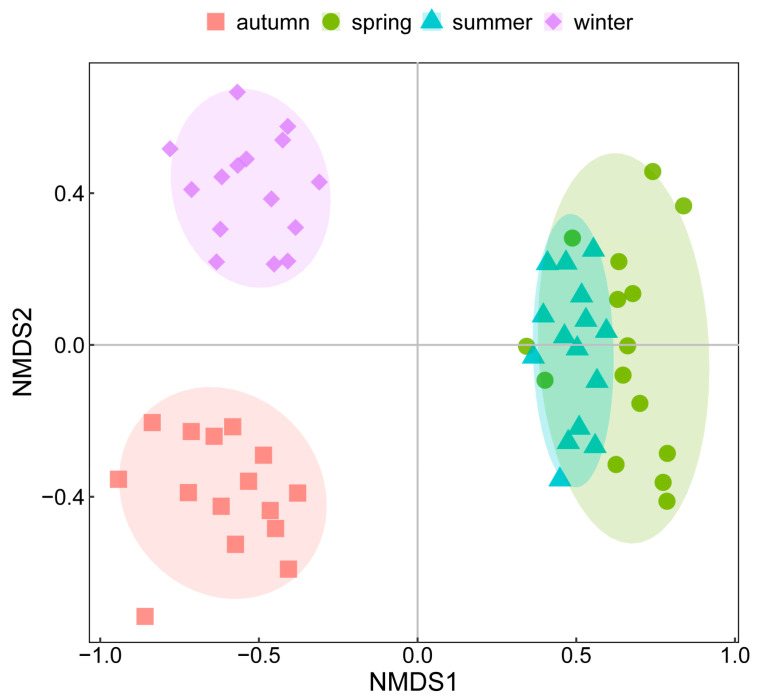
nMDS results of phytoplankton communities based on log-transformed abundance.

**Figure 4:**
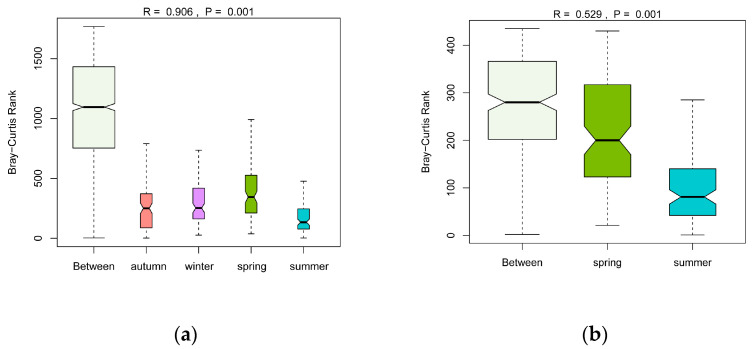
ANOSIM results of seasonal variations of phytoplankton communities: (**a**) for four seasons based on log-transformed abundance; (**b**) for spring and summer based on log-transformed abundance.

The seasonal variations of phytoplankton communities based on the Bray–Curtis distance of log-transformed abundance were visualized by nMDS with a stress value of 0.15 < 0.2 (Figure 3). Phytoplankton had noticeable seasonal variations; however, as indicated by the nMDS results, the change between spring and summer was less pronounced than the changes between other seasons. As mentioned above, the total abundance between spring and summer varied greatly, from an annual minimum of 11.57 × 10^6^ ind./L to an annual maximum of 83.13 × 10^6^ ind./L. However, the nMDS results showed a small distance between spring and summer, possibly because the nMDS weakened the dependence on the actual distance values and emphasized their rank. In other words, samples composed of different species were further apart than samples composed of the same species with great abundance differences. The ANOSIM results confirmed the significant differences among the four seasons with a high R-value of 0.906 (*p* = 0.001) (Figure 4a). The ANOSIM further analyzed the similarities of phytoplankton communities in spring and summer, and showed that R = 0.529 and *p*= 0.001 (Figure 4b), demonstrating significant discrepancies between the phytoplankton community structures in spring and summer.

### 3.3. Relationships between Environmental Factors and Phytoplankton Communities

RDA was employed to present the responses of phytoplankton abundance and biomass to environmental factors, respectively. VIF and forward-selection methods were used to select the optimal combination of environmental factors that had the greatest impact on phytoplankton abundance and biomass from 18 log-transformed and scaled environmental factors.

**Figure 5:**
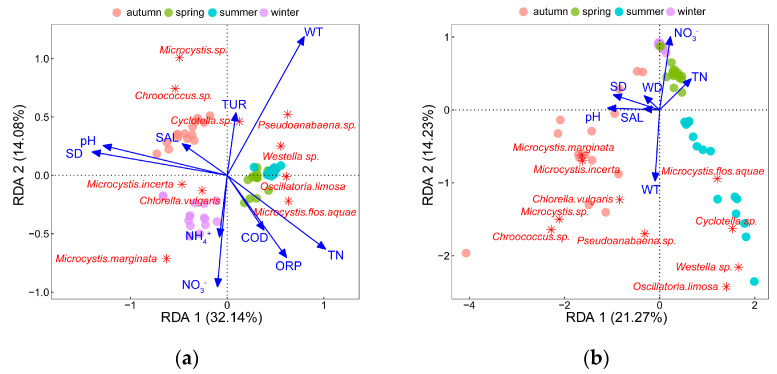

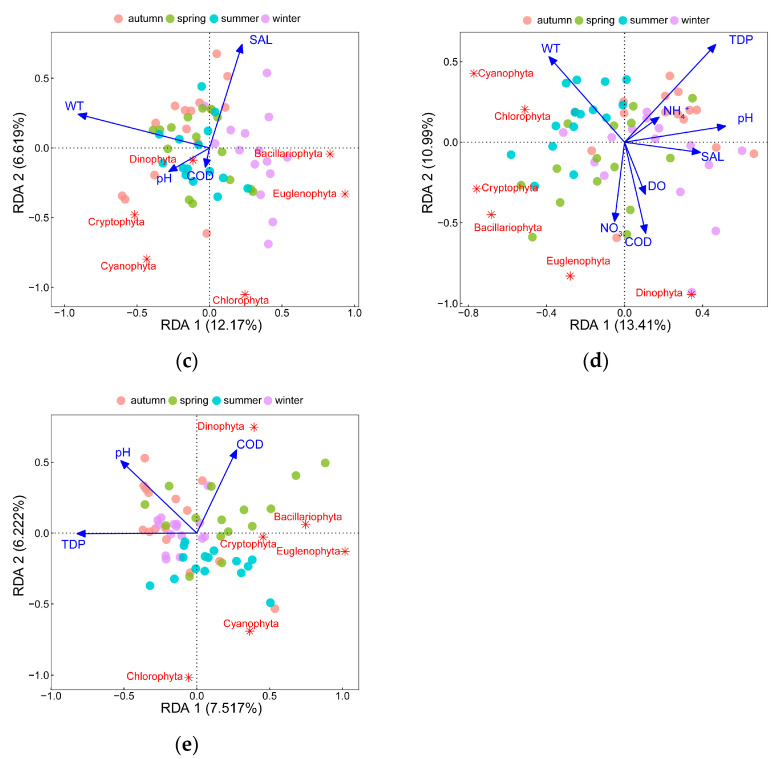
RDA of the top10 dominant phytoplankton species abundance and forward selected environmental factors: (**a**) RDA of phytoplankton species abundance; (**b**) RDA of phytoplankton species biomass; (**c**) RDA of phytoplankton species richness at phylum level; (**d**) RDA of phytoplankton community diversity at phylum level; (**e**) RDA of phytoplankton community evenness at phylum level.

For phytoplankton abundance, 10 environmental factors were filtered into the RDA model through VIF and forward-selection methods (Figure 5a), which was significantly confirmed by Mantel tests (R = 0.35, *p* < 0.001). Moreover, the first two constraint axes were meaningful at *p* = 0.009, which was also proved by Monte Carlo permutation tests (999 permutations) with Bonferroni adjustment. It explained 46.20% of the total variance of the phytoplankton abundance, with an eigenvalue of 3.21 for axis one accounting for 32.14% and 1.41 for axis two accounting for 14.06%.

As shown in Figure 5a, NO_3_^−^, WT, pH, SD, ORP, TN, COD, TUR, NH_4_^+^, and SAL significantly affected phytoplankton abundance. It could be found that *C. vulgaris* and *M. marginata* were positively associated with NO_3_^−^ and NH_4_^+^, and *M. incerta* presented notable positive correlations with SD and pH. *Cyclotella* sp., *Pseudoanabaena* sp., and *Westella* sp. were most influenced by WT. Besides this, *O. limosa*, and *M. flos-aquae* responded positively to TN and negatively to SAL. *Microcystis* sp. and *Chroococcus* sp. were negatively affected by COD and ORP.

For phytoplankton biomass, seven environmental factors were filtered into the RDA model (Figure 5b), which was confirmed to be significant by Mantel tests (R = 0.30, *p* < 0.001). Moreover, the first two constraint axes were meaningful at *p* = 0.007, which was proved by Monte Carlo permutation tests (999 permutations) with Bonferroni adjustment, and explained 35.50% of the total variance of the phytoplankton biomass, with an eigenvalue of 2.13 for axis one accounting for 21.27% and 1.42 for axis two accounting for 14.23%. Phytoplankton biomass was mainly influenced by NO_3_^−^, WT, pH, SD, TN, WD, and SAL, among which WT and NO_3_^−^ had the greatest effects as all dominant species positively correlated with WT and negatively correlated with NO_3_^−^. In addition, *M. marginata*, *M. incerta*, *C. vulgaris*, *Microcystis* sp., and *Chroococcus* sp. were positively affected by pH and SAL, while *M. flos-aquae*, *Cyclotella* sp., *Westella* sp. were negatively affected by SD.

Species richness, community diversity, and community evenness were analyzed with phylum level data (Figure 5c–e). The RDA results revealed that SAL, WT, pH, and COD could explain 18.79% of the total variance of species richness (RDA1 12.17%, RDA2 6.62%). Species richness of Bacillariophyta and Euglenophyta had negative relationships with WT, while that of Chlorophyta and Cyanophyta was negatively affected by SAL. Besides this, TDP, WT, pH, COD, NO_3_^−^, SAL, DO, and NH_4_^+^ could explain 24.4% of the total variance of community diversity, among which, TDP had strongly negative relationships with the diversity of Cryptophyta and Bacillariophyta. The diversity of Chlorophyta and Cyanophyta responded negatively to SAL and positively to WT. Additionally, COD, pH, and TDP affected community evenness obviously, where TDP was negatively associated with the evenness of Bacillariophyta, Euglenophyta, and Cryptophyta, and pH was negatively associated with the evenness of Cyanophyta. COD had a significant positive relationship with Dinophyta evenness and a negative relationship with Chlorophyta.

## 4. Discussion

### 4.1. Phytoplankton Community Composition

The temporal distribution of species abundance showed significant seasonal differences. The total abundance in autumn (79.79 × 10^6^ ind./L) and summer (83.13 × 10^6^ ind./L) was higher than in winter (14.01 × 10^6^ ind./L) and spring (11.57 × 10^6^ ind./L), which was consistent in trend with other research [41,42]. In this study, Cyanophyta, as the dominant phylum, contributed the most to the seasonal variance of total abundance, since the total abundance of Cyanophyta was highest in all seasons, especially in autumn and summer, much higher than the other phyla.

The seasonal variations in relative abundance were evident, with Cyanophyta-Chlorophyta dominating in autumn and winter and shifting to Cyanophyta-Chlorophyta-Bacillariophyta in spring and summer. Cyanophyta was dominant in all seasons, especially in autumn, showing a higher relative abundance of up to 87.78%. The results indicated that the studied area was a Cyanophyta-dominant type river from the perspective of phytoplankton. Cyanophyta became the dominant phylum in estuaries or coastal areas, as also found in many other studies [12,26,43]. Except in spring after Bacillariophyta, Chlorophyta was the second dominant phylum in the other three seasons. *C. vulgaris*, a hypersaline species, contributed the most to the dominance of Chlorophyta because it accounted for 74.59%, 88.58%, and 50.57% of the Chlorophyta in autumn, winter and summer, respectively. In this studied area, Bacillariophyta was not found to be the most prevalent phylum in all seasons as in many studies in estuaries or coastal areas [12,44,45], but it showed the same high relative abundance in spring as in other studies [42,46]. The composition of the phytoplankton community, Cyanophyta > Chlorophyta > Bacillariophyta> other, was consistent with coastal lakes in the Baltic Sea [43].

Furthermore, according to the nMDS results (Figure 3), the phytoplankton community exhibited evident seasonal variations. Even though the phytoplankton community in spring and summer had a close distance in nMDS, ANOSIM results proved a significant difference between spring and summer. The phytoplankton community showed noticeable seasonal variation rather than spatial variation, indicating that seasonality has much greater effects on the phytoplankton community than spatial influence at the annual scale (Spatial variations of abiotic factors and phytoplankton structure were shown in Supplementary Figures S1–S5 and Tables S3 and S4).

### 4.2. Response of Phytoplankton Community Characteristics to Environmental Factors

Species richness is an important index of the phytoplankton community that reflects and even determines the essential ecosystem functions [47,48,49], and is therefore widely studied in response to environmental impacts. A tendency for species richness to decrease with increasing salinity has been commonly reported, such as in the coastal Baltic Sea [21], in the Great Salt Lake, USA [20], and in the landscape water of Tianjin, China [12]. In this study, salinity also revealed a notable adverse effect on species richness, especially on Chlorophyta and Cyanophyta (Figure 5c). The salinity in the studied area was higher than 1 ppt at almost all sample stations in all seasons. It seems that that all species here were adaptable to the brackish environment. However, different species had different responses to osmotic stress variations caused by increasing salinity [50]. For example, some species could not remain to maintain normal cellular osmotic pressure and were hence eliminated [11]. On the other hand, increased salinity also influenced species reproduction. With salinity increased, the water pressure increased gradually, but some species could not produce the intracellular osmolarity needed to generate the same turgor pressure as at low salinity [50]. Additionally, increased osmotic stress would limit cell height and affect cell elongation before each cell division [51].

**Figure 6:**
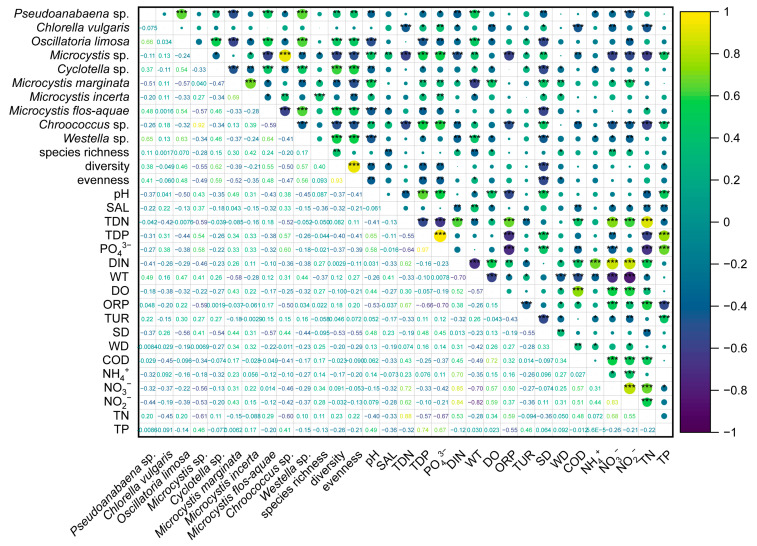
Spearman correlation analysis results of all environmental factors at the annual scale. “***” denotes *p* < 0.001, while “**” denotes *p* < 0.01 and “*” *p* < 0.05.

Righetti revealed that temperature was an important driving factor for global-scale phytoplankton diversity, and there was a nonmonotonic relationship between phytoplankton richness and temperature [18]. According to the present study, water temperature had a significant adverse effect on species richness (especially Bacillariophyta and Euglenophyta) (Figure 5c and Figure 6). This result is supported by Gruner’s experiments, which indicated that warming reduced the richness across ecosystems [52]. In the studied ecosystem here, species richness declined with increasing water temperature, which might be attributed to several factors. One was water temperature variation driven by seasonal shifts that directly led to species-richness changes. For example, the richness of Bacillariophyta and Euglenophyta had no apparent negative correlation with water temperature at the quarterly level. However, the negative relationship was notable and significant at the annual scale (four quarters taken together). This indicated that some species’ disappearances could be attributed to their physiological intolerance to high temperature [21]. Another factor is that increased water temperature induced competitive exclusion [14], indirectly leading to a decreased number of species [18]. This could explain why species richness in winter was higher than in autumn, when Cyanophyta took the absolute dominant place. In autumn, resources were taken up by fast-growing Cyanophyta. Some species were eliminated by competitive exclusion, while Cyanophyta’s dominance declined in winter. When the competitive exclusion effects decreased, other species had better growth and reproduction conditions. Additionally, although predators such as zooplankton and fish were not contained in the present study, the increasing grazing effect with water temperature could not be ignored [13].

Community diversity was mainly influenced by WT and TDP, as well as pH, SAL, COD, NO_3_^−^, DO, and NH_4_^+^ (Figure 5d). It was commonly reported that water temperature drove the dynamics of community diversity in other studies, such as estuaries in the Mediterranean Sea [53], and coastal waters during the monsoon in India [54]. In this study, WT strongly influenced the community diversity in summer and was positively correlated with the diversity of Cyanophyta and Chlorophyta. This result indicated that species of Cyanophyta and Chlorophyta were more suitable to the higher temperature in summer, and the species abundance increased with the temperature, resulting in a much more even community. Moreover, Shannon–Wiener is a classical index, reflecting the biodiversity of any population in which each member belongs to a unique group, type, or species [38], implying two potential aspects of information, abundance and species number. Combined with the influence of water temperature on species richness, a conclusion could be derived that, in summer, the positive effects of water temperature on the abundance of Cyanophyta and Chlorophyta were greater than the negative effects on species richness.

In the present study, TDP was one of the main driving factors of phytoplankton diversity, especially in autumn, and it had significant negative relationships with Bacillariophyta, Cryptophyta, and Euglenophyta. Chen indicated that phosphorus has a positive relation with blue-green algae, especially *Microcystis* [55], which further confirmed the results of this study. It was worth noting that the dominant species in autumn were all positively correlated with TDP. Additionally, many studies have found that community diversity would be decreased with the bloom of the dominant species [56,57,58]. Therefore, the lower diversity in autumn was attributed to the bloom of dominant species positively associated with TDP.

As for community evenness, it was driven by TDP, pH, and COD. The negative relationships between TDP and Bacillariophyta, Cryptophyta, and Euglenophyta coincided with the relevant correlations in community diversity, indicating that increased nutrient enrichment may favor the competitive dominance of relatively few species [20], thus leading to decreases in diversity and evenness. Inyang and Wang proposed that pH levels in coastal waters may signal organic pollution, the photosynthetic activity of phytoplankton and macroalgae, and the respiratory activity of microbial flora [22]. The adverse correlations of pH and COD with evenness was consistent with those on diversity, indicating that organism matters decreased the community diversity and evenness except for that of Dinophyta.

### 4.3. Response of Dominant Species to Environmental Factors

As Figure 5a,b show, WT, TN, NO_3_^−^, pH, and SD were the most crucial environmental factors driving the community variations in both abundance and biomass. Although salinity was also included in the RDA results through the forward-selection procedure, the effects of salinity on the abundance and biomass of dominant species were found not greater than the other environmental factors. WT was the key driving factor affecting the dominant species’ abundance and biomass, especially as dominant species’ biomass elevated with increasing WT.

*Pseudoanabaena* sp., a kind of fast-growing filamentous cyanobacteria [59], frequently occurred in cyanobacterial blooms [60,61]. It could produce not only microcystins but also an off flavor [62]. In this studied area, *Pseudoanabaena* sp. had a significant positive relationship with WT, which coincided with its high dominance values in both spring and summer, indicating it has high-temperature suitability and strong growth competitive advantage. Farnelid found that *Pseudoanabaena* sp. could contribute to N_2_ fixation in the Baltic Sea [63]. Lehtinen figured out that nitrogen-fixing phytoplankton benefited from a higher temperature [64]. Therefore, *Pseudoanabaena* sp. could satisfy its nitrogen demand through the N_2_ fixation function when the concentration of nitrate and ammonium nitrogen in the surrounding environment was low, especially at high temperature. Additionally, Li explained that the lower nitrogen concentration in higher WT was attributed to phytoplankton proliferation [65], and in the present study, the biomass of *Pseudoanabaena* sp. increased with decreasing NO_3_^−^, suggesting that *Pseudoanabaena* sp. could satisfactorily reproduce but also grow up when available nitrogen was limited.

*C. vulgaris* was found to be a hypersaline species [66] and was the dominant species except in spring partly due to the decreased salinity (3.33 ± 1.14 ppt). *C. vulgaris* became the dominant species in winter with higher NO_3_^−^ and NH_4_^+^, which indicated that the increased available nitrogen concentration was beneficial for *C. vulgaris* abundance to resist the negative effect of low temperature. However, the unfavorable relationship between NO_3_^−^ and *C. vulgaris* biomass manifested itself in the fact that the influence of WT on biomass was greater than that of nutrient concentration.

*O. limosa*, which occurred in all seasons, bloomed in spring and summer. Additionally, it was reported as an organic pollution-tolerant species with a sensitivity to pH [22], which was in accord with the negative correlation between *O. limosa* and pH, and the positive correlation between *O. limosa* and COD, ORP. Therefore, *O. limosa* could serve as an indicator of organic contamination.

*Microcystis* sp., *M. marginata*, *M. incerta*, and *M. flos-aquae.* were found not only in brackish waters [30,67], but also in marine waters with high salinity [26], as well as in fresh waters [37,61,68,69], indicating they could adapt to a broad salinity range. That these species of *Microcystis* thrived in the studied area was related to their salt-tolerant characteristic and the adaptable growth environment, such as nutrients [26], dissolved organic matter [64], and sunlight. For instance, apart from WT, *Microcystis* sp. and *M. incerta* dominated in autumn and were positively correlated with pH and SD, revealing that available sunlight and organic matter could accelerate *Microcystis* sp. and *M. incerta* proliferation. *M. marginata* had a positive response to increased NO_3_^−^ and NH_4_^+^ in winter. However, higher temperature and TN with lower pH and SD were more suitable for *M. flos-aquae.* Furthermore, although TDP was not shown in the RDA results, it was positively correlated with *Microcystis* sp., *M. marginata*, and *M. incerta* (Figure 6), demonstrating that nutrient-concentration control could be an effective measure to inhibit *Microcystis* bloom.

*Cyclotella* sp., a widespread species in both fresh and brackish water [42,70], became the dominant species in spring, in agreement with other studies [42,46]. In the present study, *Cyclotella* sp. abundance had a positive relationship with TUR, but the biomass was negatively related to SD, indicating a competitive growth advantage of *Cyclotella* sp. when the light was limited [22,37].

Redden and Rukminasari reported that *Chroococcus* sp. was abundant at all salinity levels (1.5–16 ppt) in nutrient-enriched salinity treatments [30]. In this study, it was evident that both the abundance and biomass of *Chroococcus* sp. were positively related to SAL and pH, indicating that *Chroococcus* sp. was a salt-tolerant species favored by increased pH.

As for *Westella* sp., it was commonly found in freshwater, while in this study, it occurred in four seasons and bloomed in summer, which revealed that *Westella* sp. was salinity tolerant. According to the RDA results, *Westella* sp. had a notable positive correlation with WT, suggesting that its dominance in summer was mainly driven by temperature.

## 5. Conclusions

This study was undertaken to understand the seasonal variations of the phytoplankton community and to identify the driving factors for phytoplankton community composition, species richness, community diversity, and evenness along the salinity gradient in a seagoing river, with a case study in the Duliujian River. The results showed that the studied seagoing river was of a Cyanophyta-dominant type, since the river was dominated by Cyanophyta-Chlorophyta in autumn and winter, and Cyanophyta-Chlorophyta-Bacillariophyta in spring and summer. From the perspective of driving factors, the species richness was mostly driven by WT and SAL, while WT and TDP were the most important driving factors for community diversity, as TDP, pH, and COD were for community evenness. In autumn, the dominant species bloom was positively related to the higher TDP concentration, leading to lower community diversity. In summer, the positive effects of WT on the abundance of Cyanophyta and Chlorophyta were found to be greater than the negative effects on species richness. Furthermore, seasonal variations of environmental factors resulted in different dominant species compositions, with WT being the most significant driving factor of both phytoplankton abundance and biomass. *Pseudoanabaena* sp. became the dominant species due to its strong growth ability at elevated WT, and this competitive advantage was supported by its N_2_ fixation function when nitrogen was limited. *C. vulgaris* was a hypersaline species that responded positively to NO_3_^−^ and NH_4_^+^ in winter. *O. limosa* could be an indicator of organic pollution, while *Cyclotella* sp. could indicate the degree of TUR. Finally, although more attention was needed to determine suitable environmental conditions for inhibiting phytoplankton bloom, the findings in this study could provide insights for researchers into phytoplankton community changes along the salinity gradient, and might help managers better understand the phytoplankton community’s response to environmental factors and to specify conservation measures.

## Figures and Tables

**Table 1:** The Kruskal–Wallis test results of seasonal variations of environmental factors, number of species, Shannon–Wiener index, Pielou’s evenness.

Environmental Factors	Abbreviation	Chi-Squared	df	*p*-Value
pH	pH	23.75	3	<0.0001
Salinity (ppt)	SAL	17.58	3	<0.001
Total nitrogen (mg/L)	TN	37.92	3	<0.0001
Total phosphorus (mg/L)	TP	16.72	3	<0.001
Total dissolved nitrogen (mg/L)	TDN	33.44	3	<0.0001
Total dissolved phosphorus (mg/L)	TDP	25.79	3	<0.0001
Orthophosphate (mg/L)	PO_4_^3−^	30.16	3	<0.0001
Dissolved inorganic nitrogen (mg/L)	DIN	43.85	3	<0.0001
Water temperature (℃)	WT	50.26	3	<0.0001
Dissolved oxygen (mg/L)	DO	27.78	3	<0.0001
Oxidation-reduction potential (mv)	ORP	22.87	3	<0.0001
Turbidity (NTU)	TUR	13.48	3	<0.005
Water transparency (cm)	SD	26.28	3	<0.0001
Water depth (m)	WD	8.71	3	<0.05
Chemical oxygen demand (mg/L)	COD	30.52	3	<0.0001
Nitrate (mg/L)	NO_3_^−^	44.79	3	<0.0001
Nitrite (mg/L)	NO_2_^−^	50.54	3	<0.0001
Ammonium nitrogen (mg/L)	NH_4_^+^	14.20	3	<0.005
Number of species		8.34	3	<0.05
Shannon–Wiener index	H	27.29	3	<0.0001
Pielou’s evenness	J	28.64	3	<0.0001

**Table 2:** Dominant species and dominance values for each season.

Phyla	Dominant Species	Autumn	Winter	Spring	Summer
Cyanophyta	*Pseudoanabaena* sp.	0.015	0	0.313	0.246
	*Oscillatoria limosa*	0.002	0.009	0.199	0.165
	*Microcystis* sp.	0.443	0	0	0
	*Microcystis marginata*	0.024	0.314	0	0
	*Microcystis incerta*	0.076	0.006	0	0
	*Microcystis flos-aquae*	0	0.006	0.013	0.043
	*Chroococcus* sp.	0.067	0.008	0	0
	*Oscillatoria agardhii*	0	0	0.020	0.022
Chlorophyta	*Chlorella vulgaris*	0.049	0.373	0.001	0.131
	*Westella* sp.	0	0	0.005	0.050
Bacillariophyta	*Cyclotella* sp.	0.005	0.011	0.105	0.062

## Data Availability

Data can be made available upon request to the corresponding author.

## Supplementary Materials

The following supporting information can be downloaded at: https://www.mdpi.com/article/10.3390/microorganisms11010075/s1, Figure S1: Spatial variations of salinity along the river in four seasons; Figure S2: Seasonal variations of salinity between seasons “***” denotes *p* < 0.01, while “**” for *p* < 0.05 and “*” for *p* < 0.1; Figure S3: Spatial variations of total biomass along the river in four seasons; Figure S4: Spatial variations of total abundance along the river in four seasons; Figure S5: Spatial variations of species richness along the river in four seasons; Table S1: Analytical method of water environmental factors; Table S2: Environmental factors in Duliujian River. Max = maximum values, Min = minimum values, SD = standard deviation; Table S3: Bray–Curtis distance of phytoplankton structure between stations in different seasons; Table S4: Absolute data of abiotic factors.
