# Supplementary material for: Phytoplankton Community Response to Environmental Factors along a Salinity Gradient in a Seagoing River, Tianjin, China
Source: Microorganisms. 2022 Dec 27;11(1):75. doi: 10.3390/microorganisms11010075 (PMC9864511; doi:10.3390/microorganisms11010075)
Supplement: Supplementary file 1 [file microorganisms-11-00075-s001.zip › microorganisms-2067653-supplementary.pdf]

microorganisms

Article

Phytoplankton Community Response to Environmental Factors Along a Salinity Gradient in a Seagoing River, Tianjin, China

Xuewei Sun, Huayong Zhang *, Zhongyu Wang, Tousheng Huang and Hai Huang

Research Center for Engineering Ecology and Nonlinear Science, North China Electric Power University, Beijing 102206, China
* Correspondence: rceens@ncepu.edu.cn

Supplementary Materials

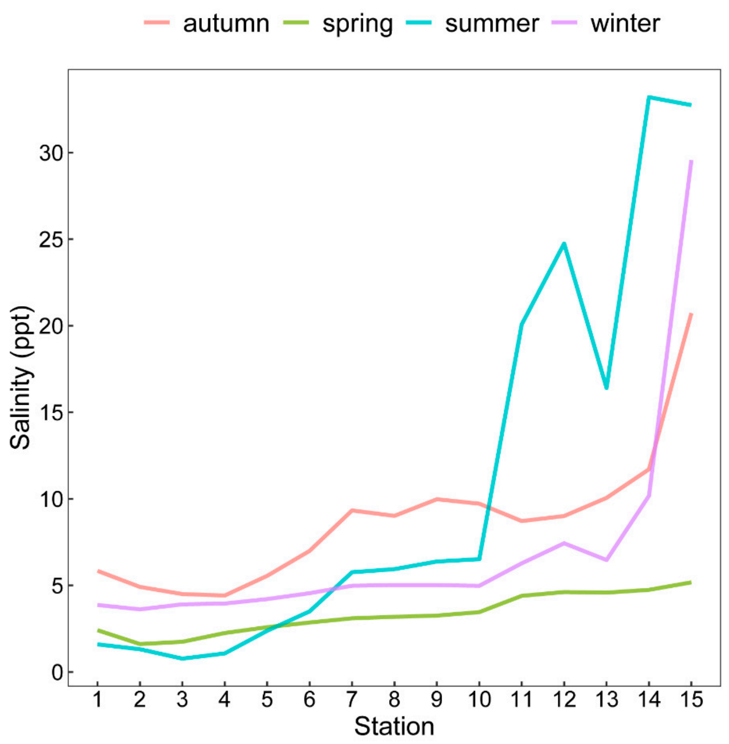

Figure S1. Spatial variations of salinity along the river in four seasons.

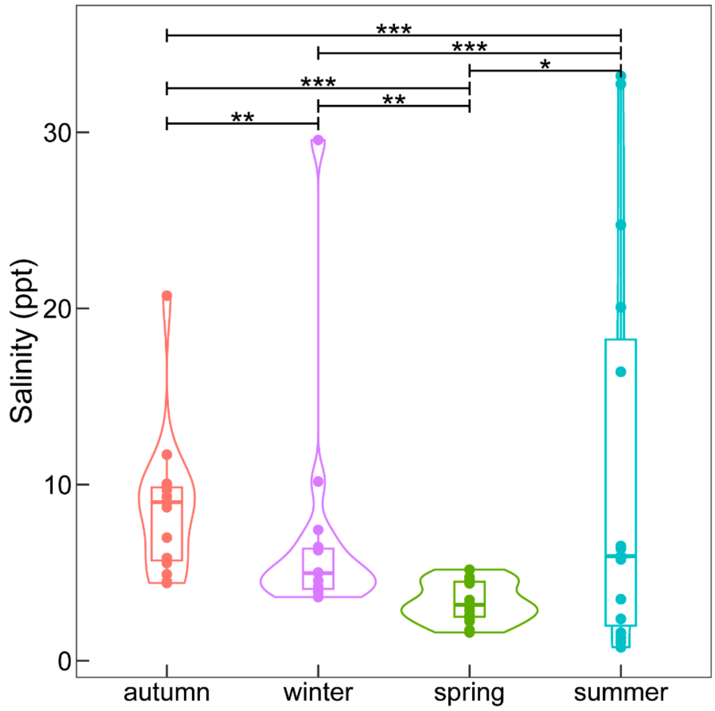

Figure S2. Seasonal variations of salinity between seasons. "***" denotes $p < 0.01$, while "**" for $p < 0.05$ and "*" for $p < 0.1$.

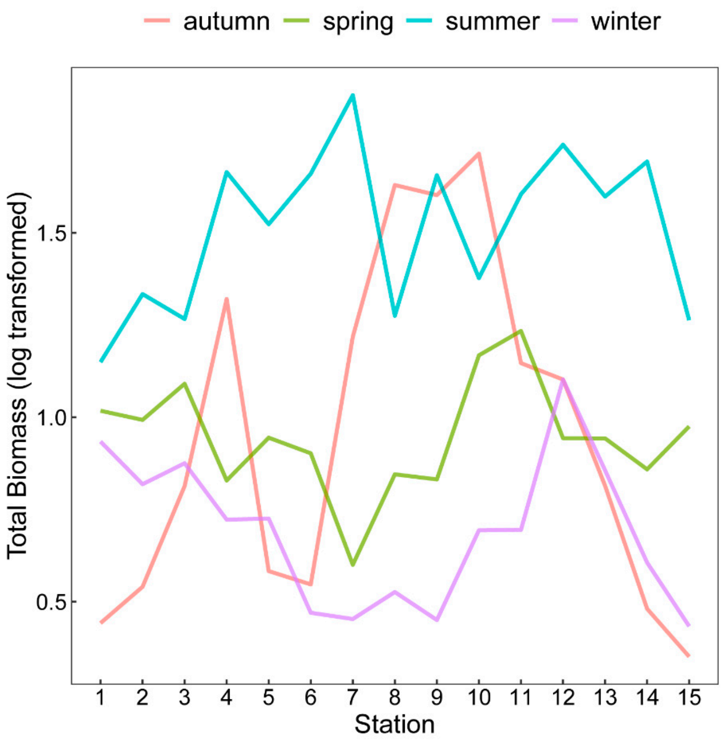

Figure S3. Spatial variations of total biomass along the river in four seasons.

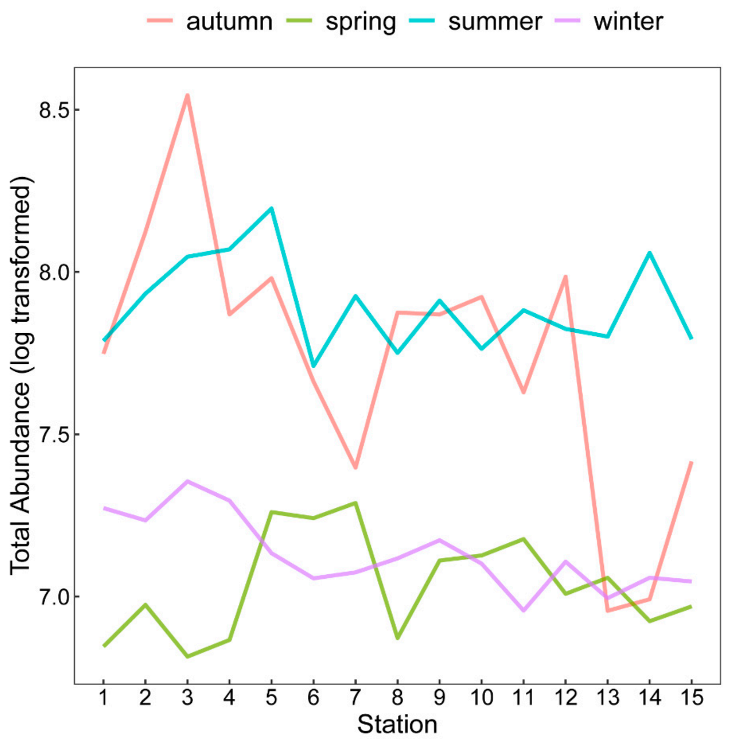

Figure S4. Spatial variations of total abundance along the river in four seasons.

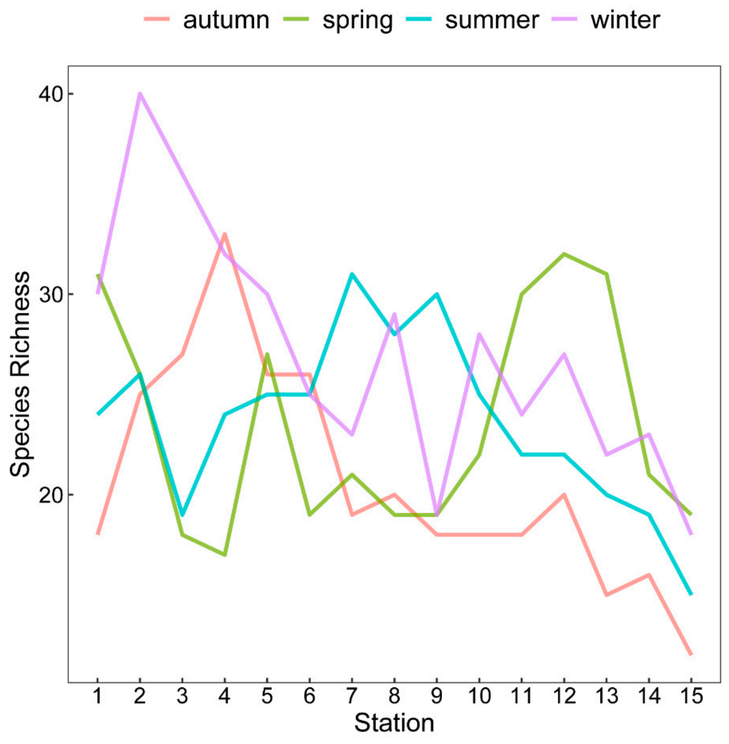

Figure S5. Spatial variations of species richness along the river in four seasons.

Table S1. Analytical method of water environmental factors.

Environmental factors	Abbreviation	Analytical method	Unit
Water temperature	WT	YSI ProPlus	℃
pH	pH	YSI ProPlus	
Dissolved oxygen	DO	YSI ProPlus	mg/L
Salinity	SAL	YSI ProPlus	ppt
Oxidation-reduction potential	ORP	YSI ProPlus	mv
Water depth	WD	Meter stick	m
Water transparency	SD	Secchi disk	cm
Total nitrogen	TN	Alkaline potassium persulfate digestion UV spectrophotometric method	mg/L
Total dissolved nitrogen	TDN	Alkaline potassium persulfate digestion UV spectrophotometric method	mg/L
Total phosphorus	TP	Ammonium molybdate spectrophotometric method	mg/L
Total dissolved phosphorus	TDP	Ammonium molybdate spectrophotometric method	mg/L
Chemical oxygen demand	COD	Dichromate method or chloride ion calibration method	mg/L
Turbidity	TUR	Hach turbidity tester	NTU
Orthophosphate	PO_4^{3-}	Molybdenum-antimony anti-spectrophotometric method	mg/L
Ammonium nitrogen	NH_4^+	Nessler's reagent spectrophotometric method	mg/L
Nitrate	NO_3^-	Gas phase molecular Absorption spectrum method	mg/L
Nitrite	NO_2^-	Gas phase molecular Absorption spectrum method	mg/L
Dissolved inorganic nitrogen	DIN	$NH_4^+ + NO_3^- + NO_2^-$	mg/L

Table S2. Environmental factors in Duliujian River. Max = maximum values, Min = minimum values, SD = standard deviation.

	Autumn				Winter				Spring				Summer			
**Environmental factors**	**Max**	**Min**	**Mean**	**SD**	**Max**	**Min**	**Mean**	**SD**	**Max**	**Min**	**Mean**	**SD**	**Max**	**Min**	**Mean**	**SD**
pH	9.73	8.65	9.16	0.39	9.19	8.39	8.92	0.21	9.13	8.35	8.78	0.19	9.10	8.08	8.53	0.28
Salinity (ppt)	20.73	4.42	8.70	4.05	29.57	3.62	6.93	6.49	5.18	1.61	3.33	1.14	33.20	0.77	10.83	11.58
Total nitrogen (mg/L)	3.11	1.41	2.03	0.57	4.19	2.28	3.13	0.52	5.11	4.00	4.59	0.26	8.07	1.69	3.44	1.80
Total phosphorus (mg/L)	0.59	0.27	0.43	0.08	0.41	0.19	0.33	0.05	0.42	0.34	0.38	0.03	0.77	0.07	0.34	0.20
Total dissolved nitrogen (mg/L)	2.71	0.92	1.19	0.44	3.06	1.75	2.20	0.42	3.70	1.87	2.88	0.40	7.03	0.97	2.13	1.83
Total dissolved phosphorus (mg/L)	0.39	0.06	0.26	0.09	0.23	0.07	0.15	0.04	0.11	0.05	0.07	0.02	0.50	0.00	0.13	0.16
Orthophosphate (mg/L)	0.32	0.05	0.23	0.07	0.19	0.04	0.11	0.04	0.06	0.01	0.02	0.02	0.46	0.00	0.10	0.16
Dissolved inorganic nitrogen (mg/L)	0.51	0.11	0.29	0.10	2.37	0.70	1.43	0.51	2.34	0.67	1.32	0.48	0.77	0.14	0.30	0.16
Water temperature (℃)	28.93	25.20	26.88	1.00	8.60	1.73	2.84	1.63	16.27	12.47	14.00	1.15	29.27	25.80	27.35	0.99
Dissolved oxygen (mg/L)	15.72	3.35	8.99	3.48	14.68	7.65	11.85	1.82	15.92	9.00	12.71	1.92	11.08	4.64	7.54	2.07
Oxidation-reduction potential (mv)	116.63	86.80	102.18	9.50	168.07	96.50	140.63	17.58	221.93	112.77	156.01	36.30	168.57	70.47	132.27	31.23
Turbidity (NTU)	77.28	7.85	35.57	22.60	34.16	11.11	15.41	5.57	36.49	14.70	20.82	6.44	54.83	12.72	27.37	13.96
Water transparency (cm)	98.83	38.83	59.74	17.95	67.33	41.67	52.96	6.79	48.67	34.00	42.80	3.93	57.00	27.00	38.84	9.42
Water depth (m)	3.99	1.28	2.65	0.71	4.42	1.12	3.09	0.85	5.46	1.43	2.81	1.01	2.99	1.28	2.25	0.57
Chemical oxygen demand (mg/L)	11.80	7.81	9.04	1.04	12.60	8.11	10.12	1.25	13.83	9.19	11.97	1.24	10.25	7.08	8.88	0.77
Ammonium nitrogen (mg/L)	0.51	0.11	0.26	0.11	1.04	0.26	0.46	0.24	1.31	0.15	0.43	0.41	0.70	0.12	0.26	0.15
Nitrate (mg/L)	0.16	0.00	0.03	0.04	1.68	0.27	0.81	0.41	1.25	0.47	0.80	0.25	0.06	0.47	0.03	0.02
Nitrite (mg/L)	0.02	0.00	0.00	0.01	0.21	0.03	0.16	0.06	0.12	0.05	0.08	0.01	0.06	0.05	0.01	0.02

Table S3. Bray-Curtis distance of phytoplankton structure between stations in different seasons.

Autumn	aut1	aut2	aut3	aut4	aut5	aut6	aut7	aut8	aut9	aut10	aut11	aut12	aut13	aut14	aut15
aut1	0.00														
aut2	0.39	0.00													
aut3	0.38	0.28	0.00												
aut4	0.53	0.49	0.50	0.00											
aut5	0.42	0.46	0.40	0.46	0.00										
aut6	0.52	0.39	0.38	0.52	0.37	0.00									
aut7	0.62	0.39	0.42	0.54	0.38	0.40	0.00								
aut8	0.49	0.49	0.42	0.48	0.58	0.50	0.43	0.00							
aut9	0.66	0.46	0.48	0.46	0.47	0.47	0.26	0.36	0.00						
aut10	0.50	0.45	0.48	0.42	0.50	0.50	0.36	0.31	0.28	0.00					
aut11	0.51	0.45	0.44	0.46	0.47	0.43	0.41	0.33	0.34	0.18	0.00				
aut12	0.53	0.57	0.49	0.48	0.49	0.45	0.50	0.32	0.42	0.38	0.28	0.00			
aut13	0.53	0.60	0.63	0.52	0.58	0.62	0.60	0.34	0.46	0.30	0.24	0.28	0.00		
aut14	0.64	0.61	0.50	0.52	0.54	0.53	0.49	0.45	0.48	0.37	0.30	0.29	0.36	0.00	
aut15	0.47	0.62	0.69	0.65	0.65	0.69	0.67	0.51	0.60	0.54	0.47	0.51	0.36	0.49	0.00
Winter	win1	win2	win3	win4	win5	win6	win7	win8	win9	win10	win11	win12	win13	win14	win15
win1	0.00														
win2	0.41	0.00													
win3	0.43	0.33	0.00												
win4	0.41	0.37	0.33	0.00											
win5	0.47	0.41	0.43	0.48	0.00										
win6	0.45	0.49	0.45	0.45	0.50	0.00									
win7	0.56	0.48	0.50	0.43	0.55	0.45	0.00								
win8	0.53	0.47	0.57	0.54	0.43	0.38	0.43	0.00							
win9	0.60	0.56	0.56	0.46	0.55	0.40	0.38	0.45	0.00						
win10	0.54	0.44	0.52	0.45	0.33	0.48	0.43	0.36	0.47	0.00					
win11	0.53	0.47	0.64	0.47	0.53	0.51	0.54	0.37	0.50	0.42	0.00				
win12	0.47	0.50	0.49	0.44	0.53	0.51	0.52	0.43	0.51	0.43	0.42	0.00			
win13	0.51	0.55	0.63	0.55	0.54	0.57	0.51	0.38	0.46	0.40	0.31	0.38	0.00		
win14	0.55	0.41	0.57	0.53	0.52	0.57	0.56	0.46	0.47	0.45	0.37	0.43	0.45	0.00	
win15	0.63	0.56	0.67	0.57	0.55	0.57	0.60	0.45	0.50	0.53	0.44	0.51	0.36	0.46	0.00
Spring	spr1	spr2	spr3	spr4	spr5	spr6	spr7	spr8	spr9	spr10	spr11	spr12	spr13	spr14	spr15
spr1	0.00														
spr2	0.33	0.00													
spr3	0.47	0.42	0.00												
spr4	0.56	0.51	0.56	0.00											
spr5	0.53	0.58	0.65	0.65	0.00										
spr6	0.48	0.51	0.62	0.53	0.48	0.00									
spr7	0.57	0.50	0.58	0.54	0.46	0.41	0.00								
spr8	0.53	0.39	0.63	0.58	0.45	0.34	0.37	0.00							
spr9	0.60	0.59	0.73	0.64	0.60	0.40	0.50	0.37	0.00						
spr10	0.53	0.51	0.56	0.61	0.51	0.45	0.54	0.48	0.42	0.00					
spr11	0.41	0.44	0.63	0.60	0.47	0.45	0.57	0.45	0.43	0.35	0.00				
spr12	0.44	0.46	0.57	0.65	0.50	0.59	0.54	0.48	0.50	0.53	0.35	0.00			
spr13	0.50	0.51	0.67	0.59	0.53	0.59	0.69	0.48	0.52	0.48	0.50	0.53	0.00		
spr14	0.43	0.40	0.60	0.58	0.66	0.64	0.62	0.50	0.59	0.59	0.50	0.57	0.46	0.00	
spr15	0.49	0.44	0.54	0.54	0.63	0.50	0.62	0.40	0.44	0.48	0.37	0.50	0.40	0.40	0.00
Summer	sum1	sum2	sum3	sum4	sum5	sum6	sum7	sum8	sum9	sum10	sum11	sum12	sum13	sum14	sum15
sum1	0.00														
sum2	0.40	0.00													
sum3	0.43	0.36	0.00												
sum4	0.45	0.25	0.27	0.00											
sum5	0.42	0.41	0.40	0.41	0.00										
sum6	0.38	0.42	0.41	0.43	0.32	0.00									
sum7	0.48	0.40	0.51	0.44	0.40	0.33	0.00								
sum8	0.36	0.39	0.60	0.53	0.44	0.33	0.24	0.00							
sum9	0.59	0.44	0.51	0.45	0.39	0.36	0.39	0.49	0.00						
sum10	0.58	0.42	0.59	0.51	0.45	0.45	0.34	0.48	0.35	0.00					
sum11	0.48	0.42	0.56	0.43	0.42	0.42	0.34	0.41	0.35	0.27	0.00				
sum12	0.56	0.42	0.52	0.40	0.46	0.45	0.37	0.49	0.43	0.32	0.23	0.00			
sum13	0.47	0.41	0.58	0.50	0.44	0.43	0.43	0.39	0.44	0.29	0.20	0.25	0.00		
sum14	0.48	0.43	0.53	0.49	0.47	0.38	0.41	0.42	0.40	0.41	0.23	0.27	0.24	0.00	

sum15	0.49	0.48	0.59	0.59	0.56	0.47	0.41	0.41	0.47	0.39	0.36	0.42	0.33	0.31	0.00

Table S4. Absolute data of abiotic factors.

Station	SAL	COD	TDP	TDN	PO_4^{3-}
aut1	5.84	1.16	0.28	0.31	5.84
aut2	4.91	1.06	0.39	0.26	4.91
aut3	4.50	0.92	0.27	0.26	4.50
aut4	4.42	0.97	0.12	0.10	4.42
aut5	5.55	1.03	0.22	0.20	5.55
aut6	6.99	1.00	0.35	0.32	6.99
aut7	9.33	1.03	0.29	0.27	9.33
aut8	9.02	1.06	0.25	0.22	9.02
aut9	9.98	0.99	0.30	0.27	9.98
aut10	9.72	1.05	0.31	0.28	9.72
aut11	8.72	1.06	0.31	0.27	8.72
aut12	9.01	1.38	0.28	0.25	9.01
aut13	10.06	1.14	0.24	0.20	10.06
aut14	11.71	1.31	0.19	0.16	11.71
aut15	20.73	2.71	0.06	0.05	20.73
win1	3.87	1.78	0.12	0.08	3.87
win2	3.62	2.23	0.14	0.10	3.62
win3	3.91	2.43	0.14	0.11	3.91
win4	3.95	3.06	0.23	0.19	3.95
win5	4.21	2.99	0.20	0.15	4.21
win6	4.55	2.65	0.20	0.14	4.55
win7	4.98	2.20	0.19	0.14	4.98
win8	5.02	2.00	0.17	0.12	5.02
win9	5.01	2.01	0.17	0.12	5.01
win10	4.98	2.00	0.16	0.11	4.98
win11	6.28	1.98	0.15	0.10	6.28
win12	7.43	1.84	0.14	0.09	7.43
win13	6.47	1.77	0.13	0.08	6.47
win14	10.18	1.75	0.10	0.05	10.18
win15	29.57	2.30	0.07	0.04	29.57
spr1	2.41	1.87	0.05	0.01	2.41
spr2	1.61	2.92	0.05	0.01	1.61
spr3	1.74	2.73	0.05	0.01	1.74
spr4	2.25	3.01	0.06	0.02	2.25
spr5	2.59	3.35	0.05	0.01	2.59
spr6	2.86	3.70	0.11	0.06	2.86
spr7	3.10	3.21	0.08	0.03	3.10
spr8	3.19	2.94	0.06	0.01	3.19
spr9	3.26	2.80	0.05	0.01	3.26
spr10	3.46	2.77	0.06	0.01	3.46
spr11	4.40	2.95	0.08	0.03	4.40
spr12	4.62	2.94	0.08	0.03	4.62
spr13	4.58	2.74	0.08	0.02	4.58
spr14	4.74	2.72	0.07	0.01	4.74
spr15	5.18	2.61	0.05	0.01	5.18
sum1	1.60	1.13	0.23	0.19	1.60
sum2	1.31	1.17	0.50	0.46	1.31
sum3	0.77	1.45	0.36	0.32	0.77
sum4	1.07	0.99	0.38	0.36	1.07
sum5	2.38	0.97	0.15	0.12	2.38
sum6	3.50	1.03	0.05	0.02	3.50
sum7	5.76	1.22	0.05	0.02	5.76
sum8	5.94	1.33	0.04	0.01	5.94

sum9	6.38	1.31	0.04	0.01	6.38
sum10	6.52	1.34	0.04	0.02	6.52
sum11	20.07	2.23	0.02	0.01	20.07
sum12	24.75	2.34	0.02	0.01	24.75
sum13	16.41	2.54	0.02	0.01	16.41
sum14	33.20	7.03	0.00	0.00	33.20
sum15	32.74	5.80	0.01	0.00	32.74
